# Current status of *L. infantum* infection in stray cats in the Madrid region (Spain): implications for the recent outbreak of human leishmaniosis?

**DOI:** 10.1186/1756-3305-7-112

**Published:** 2014-03-24

**Authors:** Guadalupe Miró, Cristina Rupérez, Rocío Checa, Rosa Gálvez, Leticia Hernández, Manuel García, Isabel Canorea, Valentina Marino, Ana Montoya

**Affiliations:** 1 Departamento de Sanidad Animal, Facultad de Veterinaria, Universidad Complutense de Madrid; 2 Asociación para la Liberación y el Bienestar Animal, ALBA, Madrid

**Keywords:** Cat, *L. infantum*, *Toxoplasma gondii*, Feline retroviruses, *Toxocara cati*, IFAT, PCR, Spain

## Abstract

**Background:**

Since 2009, the incidence of human leishmaniosis in the SW of the Madrid region has been unusually high. Although dogs are the main reservoir for this disease, a role played by dogs in this outbreak has been ruled out and investigators are now considering other hosts (eg. cats, rabbits, hares) as possible alternative reservoirs.

This study was designed to examine the *Leishmania infantum* status of stray cats in Madrid to assess its possible implications in the human leishmaniosis outbreak.

**Methods:**

346 captured stray cats were tested for antibodies against *L. infantum* by the indirect fluorescent antibody technique (IFAT) and nested-PCR methods were used to detect *Leishmania* DNA in blood samples of cats testing seropositive for *L. infantum* and/or retroviruses infection. Cats were also tested for *Toxoplasma gondii* using the direct agglutination test (DAT) and feline leukemia virus (FeLV) antigen and feline immunodeficiency virus (FIV) antibodies (PetChek* FIV/FeLV). The presence of intestinal parasites was determined using a routine coprological method.

**Results:**

The seroprevalence of *L. infantum* infection (cut off ≥ 1/100) was 3.2% (11/346). However, it was not possible to amplify *Leishmania* DNA in any of the blood samples. Seropositivity was not associated with sex, age, capture site, clinical status, retrovirus infection or *T. gondii* seropositivity. Of the 11 cats seropositive for *L. infantum*, 3 also tested positive for FIV, none for FeLV and 6 for *T. gondii*. It should be mentioned that the prevalence of FeLV p27 antigen was 4% and of FIV antibody was 9.2%. Although the seroprevalence of *T. gondii* was quite high at 53.5%, no *T. gondii* oocysts were found in any of the faeces samples analysed (n = 287). In contrast, intestinal parasites were detected in 76 (26.5%) samples, *Toxocara cati* being the most prevalent.

**Conclusions:**

Our results suggest a stable *L. infantum* infection situation among the stray cats of the Madrid area; the disease is uncommon and no clinical cases have been reported to date. The detection of other zoonotic parasites such as *T. gondii* and *T. cati* in stray cats indicates a need to adopt strict control measures in this population.

## Background

Leishmaniosis is a zoonotic disease caused by the protozoan *Leishmania infantum*. In Spain, the disease is transmitted by the female sandflies, *Phlebotomus perniciosus* and *P. ariasi*. Although dogs are considered the main reservoir, *L. infantum* has been detected in a wide range of mammalian species, including cats [1-4].

Because of its zoonotic nature, leishmaniosis has been a notifiable disease in Spain since 1982. Before 2009, the mean reported annual incidence of human leishmaniosis in the Madrid Autonomous Community (CM) was 1.12 cases/100,000 inhabitants [5]. However, this incidence increased abruptly to 22.2 cases per 100,000 inhabitants from mid-2009 to the end of 2012 in the southwest region of Madrid. In total, 446 cases were reported: 6 in 2009, 97 in 2010, 196 in 2011 and 147 in 2012 [6]. Entomological and serological surveys in the area have surprisingly detected an infection rate in dogs of 1.6-2% [7]. Other animals including cats, rats, rabbits, and hares from the affected area have also been examined. Preliminary results confirm the role that hares and rabbits could serve as wild reservoirs of leishmaniosis for the recent outbreak of visceral leishmaniosis in Madrid [8,9]. Nevertheless, antibodies against *L. infantum* have also been detected in four cats [9] and blood from cats was found in a gravid female *P. perniciosus* collected from the area affected by the outbreak [10].

In the past decade, several studies have been conducted in cats, and seroprevalences ranging from 0.9 to 59% have been reported in Mediterranean countries and Brazil [11]. In Spain, *L. infantum* seroprevalence data on cat populations is still scarce and ranges from 1.3% in the central region [4] to 28% in the South ((cut off ≥ 1/40); the real seroprevalence being 12.2% using a cut off ≥ 1:80) [1]. Prevalences detected by PCR have ranged from 0.3 to 26% for blood samples from cats in the Mediterranean area [11-13] and from 5.8 to 9.9% for bone marrow and lymph node samples from cats in Brazil [14,15].

Despite the high prevalence of canine leishmaniosis in endemic areas, feline leishmaniosis is frequently subclinical. Over 40 clinical cases in cats have been described in the literature in Europe, South America and Texas. These cases were characterized by cutaneous lesions (nodular and ulcerative lesions), enlarged lymph nodes, weight loss and ocular lesions [11,16-26]. Xenodiagnosis studies performed in two chronically infected cats have shown that a cat can be infectious to a competent *L. infantum* vector [22,27]. However, the role of the cat in the transmission cycle of leishmaniosis is not clear [28], highlighting a need for further studies.

The present study was designed to determine the status of *L. infantum* infection among the stray cats inhabiting the central region of Spain (Madrid and two of its bordering provinces Toledo and Guadalajara) and to identify risk factors associated with the presence of infection/disease. These factors include coinfection with other pathogens mainly affecting outdoor cats: feline immunodeficiency virus (FIV), feline leukemia virus (FeLV), *T. gondii* and other intestinal parasites.

## Methods

### Population studied

Every year in spring, the Animal Protection Society ALBA in Madrid undertakes a health control programme for stray cats. A large number of cats are captured to assess their health state and FeLV-FIV status and then healthy cats are sterilized before they are returned to their site of capture.

Clinical assessment consisted of sedation with a combination of 80 micrograms medetomidine/kg-5 mg ketamine/kg [29] followed by a thorough physical exam and the collection of samples: blood (serum and EDTA) by jugular venipuncture, rectum and ear swabs, and skin scrapings if cutaneous lesions were observed. Samples were kept at 4°C until processed at the laboratory.

### *Leishmania* infection

#### Serum antibody testing

For serological tests, specific antibodies to *L. infantum* were detected using the indirect immunofluorescence antibody test (IFAT) against in-house cultured promastigotes. The IFAT for anti-*Leishmania*-specific immunoglobulin G (IgG) antibodies was performed as described previously using a cut-off ≥ 1:100 to define seropositivity [4].

#### Molecular analysis

DNA was extracted from the blood samples of cats that had been identified as *L. infantum* seropositive and/or FeLV/FIV positive (n = 57) using the QIAamp DNA Mikro kit (QIAGEN). Extracted DNA was stored at -20°C until PCR was performed.

The parasite was detected using two nested PCR protocols. One was a nested-PCR targeting the *Leishmania* SSUrRNA gene (LnPCR) as described by Cruz *et al*. [30]. This protocol is *Leishmania* genus specific and uses the primer pair R221 (5’-GGTTCCTTTCCTGATTTACG-3’) and R332 (5’-GGCCGGTAAAGGCCGAATAG-3’) in the first reaction. In this second mixture, the starting primers were replaced with the primers R223 (5’-TCCCATCGCAACCTCGGTT-3’) and R333 (5’-AAAGCGGGCGCGGTGCTG-3’). The PCR product amplification size was 603 bp in the first reaction and 353 bp in the nested PCR.

The second protocol was PCR amplification of a portion of the ITS-1n gene according to the protocol described by Schönian *et al*. [31] but briefly modified. In this PCR, the region of the tandem ribosomal RNA genes (ITS-1) was amplified using the primers SAC (5'-CATTTTCCGATGATTACACC-3') and VAN2 (5'-GCGACACGTTATGTGAGCCG-3'). *L. infantum* DNA amplification was carried out in a 25 μl reaction volume containing 2.5 ul Buffer 10X (Biotools), 2 mM MgCl_2_; 0.5 ul dNTP mix 10 mM (Biotools), 0.5 ul of each primer (15 pmol/uL), 0.7 ul Tth DNA polymerase (1U/ul) (Biotools) and 10 ul of DNA. PCR amplification was performed in a thermal cycler at 80°C for 2 min, 94°C for 5 min, and 40 temperature cycles (94°C for 30 s, 57°C for 30 s, and 72°C for 30 s); this was followed by an extension step of 5 min at 72°C. The size of the PCR amplification product was 280–330 bp.

### *Toxoplasma gondii* infection

Cat sera were tested for antibodies against *T. gondii* using a direct agglutination test (DAT) kit (Toxo-Screen DA; Biomerieux) as described by Desmonts and Remington (1980). An antibody titre of 1:40 was considered indicative of exposure of the cat population to *T. gondii*[32].

### FeLV-FIV infection

Cats were tested for FeLV antigen (p27) and FIV antibodies using a commercial ELISA kit (PetChek* FIV/FeLV; IDEXX Laboratories) [33].

### Other parasitological methods

Faeces samples were collected using a rectal swab. For coprological analysis, we used the modified Telemann sedimentation method plus merthiolate-iodine-formalin staining, followed by examination under a light microscope [34].

Ear swabs were also collected to determine the presence of ectoparasites, mainly *Otodectes cynotis*. Auricular secretions were examined under the microscope.

Whole skin was explored to detect the presence of skin lesions and during this process other ectoparasites were sometimes detected. When these were detected, they were stained in 70° alcohol until their identification under the microscope and/or magnification using identification keys [35,36].

### Statistical analysis

Correlations between all the variables examined were identified by the Chi-square test (SPSS 17.0). Significance was set at *p* < *0.05*.

### Ethical considerations

The study was carried out in accordance with Spanish Legislation guidelines (Ley 1/1990, Comunidad de Madrid) and the International Guiding Principles for Biomedical Research Involving Animals, issued by the Council for the International Organizations of Medical Sciences.

## Results

Of the 346 stray cats (146 male, 200 female) included in the study, 181 were enrolled in 2012 and 165 in 2013. Cats were grouped according to age: 6.3% (22/346) were kittens (under 6 months), 25.7% (89/346) were young (6 months to 1 year) and 67.9% (235/346) were estimated to be older than one year.

**Figure 1:**
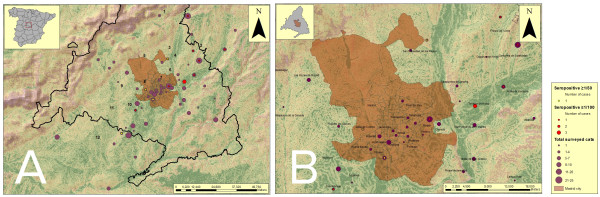
**Geocoded sites where the cats were captured, shown on a digital elevation model. A**. Distribution of cats seropositive for *L. infantum* (IFAT titre ≥ 1:50) in the study area. **B**. Detailed distribution of cats seropositive for *L. infantum* (IFAT titre ≥ 1:50) in the Madrid city area. 1: Uceda (Guadalajara); 2: Guadalajara city; 3: Fresno del Torote (CM); 4: Camarma de Esteruelas (CM); 5: San Sebastián de los Reyes (CM); 6: Alcalá de Henares (CM); 7: Torrejón de Ardoz (CM); 8: Las Rosas (Madrid city); 9: Casa de Campo (Madrid city); 10: Orcasur (Madrid city); 11: Fuenlabrada (CM); 12: Carranque (Toledo). **For 3 seropositive cats (two showing a titre of 1:50 and 1 of 1:100), the capture site was unknown so they do not appear on the map.

The cats were captured throughout the Madrid region (Madrid Autonomous Community, CM) and its bordering provinces: 25 cats in Guadalajara (NW of CM), 38 in Toledo (S of CM) and 272 in CM (Figure 1). Of these 272 cats captured in the Madrid region, 123 were found in the city, 57 in the eastern part of the region, 20 in the northeastern part, 16 in the northwestern part, 30 in the southwestern part and 26 in southeastern Madrid. The capture sites for 11 cats were unknown (Table 1).

**Table 1:** ***L*. *infantum *** **infected cats according to sex, age, capture site and clinical signs compatible with leishmaniosis**

**Variables**	**Total**	***L*. *infantum *** **IFAT**		***p*-*value***
		**Positive (%)**	**Negative (%)**	
**Sex**				*0.14*
Male	146	7 (4.8)	137 (95.2)	
Female	200	4 (2)	196 (98)	
**Age**				*0.52*
< 6 months	22	0 (0)	22 (100)	
6 months- 1 year	89	2 (2.2)	87 (97.8)	
> 1 year	235	9 (3.8)	226 (96.2)	
**Clinical signs**				*0.63*
Absence	326	10 (3.1)	316 (96.9)	
Presence	20	1 (5)	19 (95)	
**Capture site**				*0.13*
Toledo	37	0 (0)	37 (100)	
Guadalajara	25	2 (8)	23 (92)	
Madrid				
City	123	2 (1.6)	121 (98.4)	
Northeast	20	1(5)	19 (95)	
East	57	5 (8.8)	52 (91.2)	
Southeast	26	0 (0)	26 (100)	
Southwest	30	1 (3.3)	29 (96.7)	
Northwest	16	0 (0)	16 (100)	
Unknown	11	0 (0)	11(100)	
**Total**	346	11 (3.2)	335(96.8)	

In a clinical examination, 326 cats were classed as healthy and 20 cats had clinical signs compatible with feline leishmaniosis. The clinical signs more frequently observed were skin lesions (alopecia and crusts), ocular lesions (eg. conjunctivitis, keratitis), weight loss and lymphadenomegaly.

The distribution of seropositive cats by capture site is shown in Figure 1. *Leishmania infantum* seroprevalences were 3.2% (11/346) as determined by IFAT (cut off ≥ 1:100): observed titres were 1:100 (n = 4) (sites 2, 3, 7, and one unknown), 1:200 (n = 5) (sites 4, 6, 7 (n = 2) and 8) and 1:400 (n = 2) (sites 1 and 11) (see Figure 1A for sites). In a further six cats, antibody titres were 1:50. This meant a percentage of cats with antibodies against *L. infantum* of 4.9% (17/346). Correlations between the seroprevalence data and sex, age, clinical signs and capture site are shown in Table 1. No significant differences in seroprevalence emerged according to these factors.

Blood samples from 57 cats testing seropositive for *L. infantum* and/or positive for feline retroviruses were subjected to PCR to detect the presence of *L. infantum* DNA. However, none of these tests proved positive.

Feline retroviruses (FeLV-FIV) were detected in 43 cats: 14 tested positive for FeLV infection, 32 for FIV and 3 cats were positive for both retroviruses.

Antibodies against *T. gondii* were detected in a high proportion of the animals (185 cats, 53.4%). However, *T. gondii* oocysts were not observed in faeces samples.

Of the 11 cats that were seropositive for *L. infantum*, 3 tested positive for FIV, zero for FeLV and six for *T. gondii*. No significant correlations were detected between *L. infantum* and FIV (*p* = *0.11*), *L. infantum* and FeLV (*p* = *0.48*) or *L. infantum* and *T. gondii* (*p* = *0.94*) (Table 2).

**Table 2:** ***L*. *infantum *** **infected cats and co-infection with feline immunodeficiency virus (FIV), feline leukemia virus (FeLV) or *Toxoplasma gondii***

		***L*. *infantum *** **IFAT**		***p*- *value***
		**Positive (%)**	**Negative (%)**	
**FIV**	Positive	3 (27.3)	30 (9)	*0.11*
	Negative	8 (72.7)	306 (91)	
**FeLV**	Positive	0 (0)	14 (4.2)	*0.48*
	Negative	11 (100)	321 (95.8)	
***T*. *gondii***	Positive	6 (54.5)	179 (53.4)	*0.94*
	Negative	5(45.5)	156 (46.6)	

Faecal samples were collected from 287 cats and intestinal parasites were detected in 76 (26.5%) of these samples. The intestinal parasites observed were: *Toxocara cati* (7.7%), *Taenia* spp. (6.9%), *Cystoisospora* spp. (6.3%), *Giardia duodenalis* (2.4%), *Joyeuxyella* spp. (1.7%), *Dipylidium caninum* (1.4%) and *Aelurostrongylus abstrusus* (0.3%).

In ear swab samples collected from 281 cats, 36 (12.8%) showed the presence of *Otodectes cynotis*. In some cats, other ectoparasites were also detected during physical examination: *Ctenocephalides felis* (n = 13), *Felicola subrostratus* (n = 2), *Rhipicephalus sanguineus* (n = 1) and *Demodex* spp (n = 1).

## Discussion

In this study, the seroprevalence of *L. infantum* in stray cats in Madrid and its neighbouring provinces was estimated at 3.2%. Prior studies have indicated similar seroprevalences for Madrid (1.3-3.7%) [3,4] and other Mediterranean regions such as Greece [37], Portugal [38,39], southern Italy [40] and Israel (Jerusalem) [41]. In contrast, reported seroprevalences for Ibiza (13.2%) [42] and southern Spain (28.3%) [1] have been notably higher. These two regions are endemic for canine leishmaniosis and seroprevalences of *L. infantum* in dogs are also much higher than in other Spanish regions [43-45]. We should, however, mention that these rates are difficult to compare due to differences in the diagnostic techniques used (IFAT, ELISA), the cut-offs established, the sizes and origins of samples, and the season of study. There is a clear need to standardize protocols, establish cut-offs for the different techniques and define a gold standard procedure, as in the case of canine leishmaniosis [46,47].

Despite these differences among the seroprevalence studies performed to date, the seroprevalence of *L. infantum* among cats observed here and in other studies has not substantially changed since the first studies conducted in Madrid [3,4]. This trend has been paralleled by the *L. infantum* situation in dogs in Madrid, where the seroprevalence over the past 15 years has been stable at 6.4 to 8.1% [43,48,49], including the time period corresponding to an unusual outbreak of human leishmaniosis in SW Madrid [6].

Although PCR is a sensitive method [50], we were unable to detect *L. infantum* DNA in any of the blood samples testing seropositive for the pathogen. One possible reason for this inconsistency is that blood is not a good sample for this purpose or that the sample volume generally used is insufficient (100 μl) [2,51]. Similar studies have also reported a low prevalence of *Leishmania* positivity detected by PCR in blood samples (0.3-0.6%) collected from cats in Madrid and N Portugal, where the seroprevalence is also lower (2.8-3.7%) [3,12,52]. Surveys in the Balearic Islands, S Spain and S Portugal have detected higher percentages of cats testing positive for *Leishmania* infection by PCR on blood samples (8.7-26%) [13,38,42,53]. In these studies, however, seroprevalences were also higher (13.2-26%) [1,42]. The findings of molecular studies from Brazil are not comparable to these data because the samples used for PCR were bone marrow, spleen and lymph nodes [14,15,54]. This type of sample probably should be the sample of choice yet has the drawback that invasive methods are needed for their collection. Also, with cats being smaller and more difficult to handle than dogs, samples are difficult to obtain. These limitations prompt a need to assess the sensitivity of the use of non invasive samples (eg. conjunctival swabs), as reported for canine leishmaniosis [55], for the PCR detection of *Leishmania*. Further molecular studies need to determine whether samples such as bone marrow, lymph nodes, conjunctival/oral swabs and/or skin will improve the detection of *Leishmania* DNA and diagnosis of the parasite [14,42,51].

In the current study, no significant correlation was found between *L. infantum* infection and sex, age or clinical status, as reported in similar studies [1,41,56]. According to the capture site, seroprevalences were higher in the E (8.8%) and NE of Madrid (10%) and in Guadalajara (8%), though differences were not significant (*p* = *0.13*). Cats seropositive for *L. infantum* showed a homogenous distribution in the area of the human leishmaniosis (SW) outbreak, indicating that cats are not playing a role as a reservoir for *L. infantum* infection in this area.

Reported clinical cases of feline leishmaniosis are frequently associated with skin lesions as well as other less specific clinical signs (eg, lymph node enlargement, weight loss, ocular involvement), which can also be observed in other infectious diseases such as those produced by retroviruses. Accordingly, most cats developing clinical signs are suspected of having an impaired immune system [11]. In our study, 5.7% (20/346) of the cats showed clinical signs compatible with feline leishmaniosis, of which, only one was seropositive for *L. infantum* antibodies. These results are in line with those of prior studies in which high proportions of seropositive dogs and/or cats showed no clinical signs of leishmaniosis [13,14,42,48] and suggest the need for tests (e.g. serology, molecular diagnosis, etc.) in companion animals regardless of the presence of clinical signs, since in many cases the disease is underdiagnosed.

The prevalence of feline retroviruses (FeLV/FIV) in stray cats of our study area was consistent with the rates detected in previous studies [2,42]. FeLV and FIV infections are frequently associated with opportunistic infections caused by protozoans, fungi, bacteria or viruses since these viruses may compromise the cellular immune response [57]. Sobrinho *et al*. [14], and Pennisi *et al*. [40] reported feline leishmaniosis in association with FIV, while in a study performed by our group in Ibiza (Spain) association between *L. infantum* and FeLV was detected [42]. In the present study, certain positive association was observed between *Leishmania* and retroviruses as reported in Jerusalem (Israel) [41] and Brazil [56]. However, since leishmaniosis is an immunomediated disease [58], future studies need to address possible correlations with immunosuppressive diseases or states (eg, retrovirus infections, neoplasias, treatment with corticosteroids, etc.) [2,11].

Antibodies against *T. gondii* were detected in 53.4% of the stray cat population studied. This seroprevalence is similar to that reported by Aparicio *et al*. [59] and Alonso *et al*. [60] for stray cats in Madrid, though we previously observed a lower seroprevalence over the period of 2003–2007 [61,62]. The presence of cats testing seropositive for *T. gondii* is significant because, despite not detecting the oocysts of *T. gondii* in any faeces sample, these cats must have excreted oocysts sometime during their life, mainly when they were young. This could not be confirmed, however, due to the low numbers of kittens and young cats included in our study. Thus, the oocysts of *T. gondii* and other zoonotic parasites such as *Giardia duodenalis* and *Toxocara cati*[63], also detected here, could be present in the soils of public parks. In effect, *Toxocara cati* was the intestinal parasite appearing at the highest prevalence. *Toxocara* spp. (*Toxocara cati* and *T. canis*) are responsible for visceral larvae migrans (VLM) in humans and although, *Toxocara canis* is often more recognized as a zoonotic agent, *Toxocara cati* should not be overlooked [64,65]. Moreover, some patients with ocular larva migrans (OLM) due to *Toxocara* spp. show a stronger reaction to the *T. cati* than *T. canis* antigen in the Ouchterlony test [64]. It appears that *T. cati* may contribute to OLM and VLM in larger measure than was previously thought [64,65]. The prevalence of the other intestinal parasites was similar to that detected in stray cats from other major cities such as Lisbon [39] or Milan [66] or even in other studies performed in Madrid [61,62].

Prevalences of the ectoparasites *Otodectes cynotis*, *Ctenocephalides felis*, *Rhipicephalus sanguineus* and *Felicola subrostratus* were similar to those reported by others [39,67]. As vectors for zoonotic diseases (eg, dipylidiosis, ricketsiosis, bartonellosis, etc.), many of these ectoparasites will have implications for public health.

## Conclusions

In conclusion, the results of this study point to the stable situation of *Leishmania infantum* infection among the stray cats of Madrid and neighbouring areas (Toledo and Guadalajara), with clinical cases being rare. Further molecular and xenodiagnosis studies are, however, needed to clarify the epidemiological role of this alternative host. We suggest that veterinarians in canine leishmaniosis endemic areas should include leishmaniosis in the differential diagnosis of cats with compatible cutaneous or systemic clinical signs and inform cat owners of measures to prevent or reduce disease transmission. In addition, efficient repellents/insecticides to prevent sandfly bites need to be developed for use in cats.

The detection of other important zoonotic parasites such as *Toxoplasma gondii*, *Toxocara cati*, *Giardia duodenalis*, etc. in the stray cats analyzed identifies a need for appropriate control measures in this population.

## Competing interests

The authors declare they have no competing interests.

## Authors’ contributions

GM conceived and coordinated the study, and participated in its design, the field study and drafted the manuscript. CR and MG participated in the physical exam and collection of blood samples from cats. RC participated in collecting samples and carrying out the molecular technique. RG participated in data elaboration and helped to draft the manuscript. LH and VM participated in the diagnostic assays. IC participated in the molecular assays. AM participated in the field studies and the diagnostic assays, performed the statistical analysis, and helped draft the manuscript. All authors read and approved the final manuscript.
